# Common Vetch, Valuable Germplasm for Resilient Agriculture: Genetic Characterization and Spanish Core Collection Development

**DOI:** 10.3389/fpls.2021.617873

**Published:** 2021-03-09

**Authors:** Lucía De la Rosa, María Isabel López-Román, Juan M. González, Encarnación Zambrana, Teresa Marcos-Prado, Elena Ramírez-Parra

**Affiliations:** ^1^Centro de Recursos Fitogenéticos, Instituto Nacional de Investigación y Tecnología Agraria y Alimentaria, Alcalá de Henares, Spain; ^2^Centro de Biotecnología y Genómica de Plantas, Instituto Nacional de Investigación y Tecnología Agraria y Alimentaria, Universidad Politécnica de Madrid, Pozuelo de Alarcón, Spain; ^3^Departamento de Biomedicina y Biotecnología, Universidad de Alcalá, Alcalá de Henares, Spain

**Keywords:** *Vicia sativa*, common vetch, genetic diversity, core collection, Plant Genetic Resources, genebank, legume

## Abstract

Common vetch (*Vicia sativa* L.) is a legume used for animal feed because of its high protein content and great capacity for nitrogen fixation, making this crop relevant in sustainable agriculture. The Spanish vetch collection, conserved at the Spanish Plant Genetic Resources Center (CRF), is one of the largest collections of this species worldwide, including landraces, wild relatives mainly collected in Spain, and commercial cultivars, but also accessions of international origin. The analysis of the genetic diversity of this material, whose genome has not been sequenced yet, and the assembly of a representative collection could play a pivotal role in conserving and exploiting these genetic resources in breeding programs mainly in those focused on consequences and demands of climate change. In this work, a set of 14 simple sequence repeat (SSR) reference alleles for genetic diversity analysis of the CRF vetch collection has been developed, used for genotyping more than 545 common vetch accessions from all over the world and validated. All the tested markers were polymorphic for the analyzed accessions. Overall, at least 86 different loci were identified with 2–11 alleles per locus with an average of 6.1 alleles per locus. Also, the analyses of the generated SSR database support that most of these SSR markers are transferable across closely related species of *Vicia* genus. Analysis of molecular variance revealed that wild relatives have a higher genetic diversity than landraces. However, cultivars have similar diversity than landraces, indicating that genetic variability has been barely lost due to the breeding of this legume. Low differences of genetic variations between Spanish and non-Spanish accessions have been observed, suggesting a high degree of diversity within Spanish genotypes, which provide 95% of the total genetic variation, so we have focused our efforts on characterizing genotypes of Spanish origin that were further studied using storage protein profiles. Based on SSR, seed protein profiles, and agromorphological and passport data, a vetch core collection (VCC) containing 47 *V. sativa* accessions of Spanish origin has been established. In this collection, the characterization has been expanded using ISSR markers, and it has been reevaluated with new agromorphological data, including drought tolerance characters. This VCC presents a minimum loss of genetic diversity concerning the total collection and constitutes an invaluable material that can be used in future breeding programs for direct use in a resilient agricultural system.

## Introduction

The common vetch (*Vicia sativa* L., Tribe *Viciae*, Family *Fabaceae*) is one of the world’s most economically important annual grain legume, consumed also as forage. Common vetch is mainly used for animal feed as a cheap and rich source of protein and minerals of high digestibility and high energy content (Huang et al., 2017). Furthermore, vetch fixes atmospheric nitrogen through its symbiotic interactions with rhizobia soil bacteria, so its cultivation is appropriate in sustainable agricultural systems by decreasing the use of fertilizers and reducing CO_2_ emissions and other pollutants (Dalias and Neocleous, 2017). Archeological evidences suggest that the Mediterranean basin is the center of origin and primary diversification (Zohary and Hopf, 2000; Maxted and Bennett, 2001). However, because of its economic and ecological advantages, vetch is now widespread throughout many parts of the world. Food and Agriculture Organization of the United Nations last data, corresponding to the year 2018, indicate that global vetch production is mainly concentrated in Ethiopia, the Russian Federation, and Spain. Worldwide, total vetch production is more than 934,388 tons/year, with a crop area of 540,761 ha^1^.

Although the total number of varieties and accessions of common vetch is quite difficult to estimate, most of the genetic resources of *V. sativa* are maintained *ex situ* in germplasm collections. GENESYS, the online platform that includes information on Plant Genetic Resources (PGR) for food and agriculture conserved in genebanks worldwide^2^, includes information of 13,337 accessions named as *V. sativa*, mainly traditional cultivars (landraces) mostly collected in Russia, Turkey, Spain, Italy, Syria, and Bulgaria. EURISCO, the European catalog of PGR^3^, includes a total of 8,101 accessions with the name of *V. sativa*. The most important collections are those kept in the Russian Federation, Germany, Spain, and Bulgaria. Genebanks are crucial resources for the conservation of natural genetic diversity and provide a source of novel features for fluctuating circumstances including climate change and new disease outbreaks and are essential for sustained crop improvement (Ramírez-Villegas et al., 2013; Pellegrini and Balatti, 2016; Dempewolf et al., 2017). Nevertheless, the main limitation of using these PGR collections is the lack of easily available characterization and evaluation data (Van Hintum and Brink, 2019), essential for analyzing their genetic diversity, identifying potential valuable traits, and selecting local varieties that could be used directly by farmers or incorporated into breeding programs (Hodgkin et al., 2003; Westengen et al., 2018). The use of agromorphological descriptors has been the classical approach in PGR characterization and evaluation, but the cost of these trials, both in monetary input and human labor, and the strong environmental influence of these variables limit their use (Rivera et al., 2018). With the development of molecular marker technology, multiallelic and locus-specific codominant markers have been used for many applications in genetic evolution and diversity studies, comparative genomics, cultivar identification, quantitative trait locus (QTL) identification, linkage mapping, and molecular assisted breeding (Collard and Mackill, 2008). Recent efforts have been done toward the development of markers from coding regions, acting as potential “functional genetic markers,” which may allow the tagging of QTLs for relevant agronomic important traits. Furthermore, comparing with markers from non-coding regions, these markers are likely to be better conserved and may be more transferable across closely related species (Collard and Mackill, 2008).

A recent analysis of genome size in *Fabaceae*, including *V. sativa*, reveals a genome size of 1.77 Gbp/1C, which has not been sequenced (Macas et al., 2015). In fact, a deficiency of publicly available genomic resources in *V. sativa* has restricted the efforts to improve this crop. Previous works have analyzed the genetic diversity of *Vicia* genus employing retrotransposon-based SSAP markers (Sanz et al., 2007) and *V. sativa* associated with seed storage protein profiles in a collection of Spanish underground vetches (De la Rosa and González, 2010), the genetic singularity coefficients using AFLP markers in Russian varieties (Potokina and Aleksandrova, 2008) and intrapopulation diversity of four common vetch varieties using start codon targeted marker methodology (Chai et al., 2017). Recent efforts in transcriptome analysis using next-generation sequencing (NGS) technology, not only facilitates gene expression profiling and genome annotation in a high-throughput, relatively cost-effective way, but also provides an extremely useful source for molecular markers, such as single-nucleotide polymorphisms (SNPs) and simple sequence repeats (SSRs), which may be associated with functional transcribed genes (Edwards and Batley, 2010; Kumar et al., 2017). Microsatellites or SSRs are considered of great efficiency because they combine high genome coverage, relative abundance, polymorphism, reproducibility, and codominant nature (Vieira et al., 2016). The recent availability in the genebank databases of several expressed sequence tags (ESTs) has allowed the identification of microsatellite-type molecular markers with different degrees of polymorphisms in *V. sativa* (Chung et al., 2013; Liu et al., 2014; Zhu et al., 2019).

The Spanish vetch collection, conserved at the National Center for Plant Genetic Resources (CRF-INIA), is one of the largest in the world and includes landraces, wild relatives, and commercial cultivars from both Spanish and international origin. The main goal of this work has been the characterization and evaluation of the diversity of this collection using a novel set of SSR-type molecular markers specifically designed and validated for this plant species. Moreover, this minimal standard SSR set of 14 reference alleles from *V. sativa* has also been validated by extrapolating their use in the characterization of other *Vicia* species.

One of the main troubles for germplasm conservation, regeneration, duplication, and documentation is the large size of many collections (Van Hintum et al., 2000). One way to solve this problem is to establish a core set of accessions that represent the diversity of the collection in a reasonable number of individuals. Thus, another important aim of this article has been the establishment of a vetch core collection (VCC) of the diversity of genotypes focusing our interest on the study of accessions of Spanish origin. For this goal, in addition to the SSR, seed storage protein profiles, agromorphological markers, and passport data have been used. The study of VCC was expanded using Inter-Simple Sequence Repeat (ISSR)-type molecular markers and new agromorphological data that will include some relevant agronomic traits, including drought-associated parameters. The main interest of the construction and characterization of this Spanish common VCC would be to establish a primary genetic resource for common vetch breeding.

## Materials and Methods

### Plant Materials and Genomic DNA Extraction

The 545 *V. sativa* accessions analyzed in this study are conserved at the CRF, and their passport data are available at https://bancocrf.inia.es/en/. The correspondences between the code used at the National Inventory and those used in this work are indicated in Supplementary Table 1. Other accessions of *Vicia* genus, including different species, are described in Supplementary Table 2. Supplementary Tables 1, 2 include additional information on taxonomy details, passport data, and biological status.

For genomic analysis *V*. *sativa* seeds were germinated and grown in greenhouse (16-h/8-h light–dark cycle, at 22°C), and young leaf samples from one plant of each accession were collected from 4-week-old plants and preserved at −80°C until genomic DNA extraction. Genomic DNA was extracted using a DNeasy^®^ Plant Mini kit (Qiagen, Spain) according to the manufacturer’s instructions. DNA quantity and quality were analyzed using a NanoDrop ND1000 instrument (Thermo Fisher Scientific).

### SSR Genotyping: Multiplex Polymerase Chain Reaction Amplification and Data Processing

The 14 selected pairs of oligonucleotides containing SSR were validated experimentally, and forward primers were synthesized with the addition of different fluorescent probes (Dye Set DS-30: 6-FAM, HEX, NED, PET, LIZ) as indicated (Supplementary Table 3). Polymerase chain reaction (PCR) amplification was performed in a 12-μL reaction volume containing 20 ng of genomic DNA, using Multiplex PCR NZYTaq 2x Colourless Master Mix (NZYTech Genes & Enzymes, Portugal) according to the manufacturer’s instructions. The cycling PCR parameters were one cycle: 94°C for 5 min and 10 cycles: 94°C for 30 s, 50°C for 30 s, 72°C for 45 s; 25 cycles: 94°C for 30 s, 54°C for 30 s, 72°C for 45 s, followed by a final extension at 72°C for 7 min. PCR fragments obtained containing SSR alleles were resolved on an ABI 3130xl Genetic Analyzer (Applied Biosystems). Fragment-size determination and data analysis were determined by using Peak Scanner 2.0 Software (Applied Biosystems). Electrophoresis patterns were recorded, followed by manual scoring of the peaks. An internal size standard (GeneScan1200-LIZ; Applied Biosystems) was used for exact size determination, rounding up or down the decimal places to reach integer allele numbers. The fragment size data were used to construct a codominant data matrix that was used for further analysis of genetic diversity, population structure, and phylogenetic analysis.

### ISSR Genotyping

ISSRs were PCR-amplified in 15-μL volume containing 20 ng genomic DNA following the procedures previously described (Hammami et al., 2014). A total of 24 sets of primers were tested. Ten of them that produced unambiguous and reproducible fragments were selected (Supplementary Table 4). All experiments were performed in triplicate. The cycling PCR parameters were one cycle: 94°C for 5 min; 40 cycles: 94°C for 30 s, 52°C for 45 s, 72°C for 2 min, followed by a final extension at 72°C for 7 min. The amplified products were loaded onto 1% (wt/vol) agarose gels and separated by horizontal electrophoresis using a 1× TAE and 100 V/cm. The gels were then dyed with ethidium bromide, photographed using an Alpha Image EC image capture, and analyzed by AlphaView software.

### Analysis of Seed Storage Proteins

Seed storage proteins were analyzed by one-dimensional polyacrylamide gel electrophoresis in the presence of sodium dodecyl sulfate (SDS) polyacrylamide gel electrophoresis. Seed protein extracts were prepared by removing the seed coat and then mixing 20 mg of cotyledon meal with 500 μL of extraction buffer containing 2% (wt/vol) SDS, 0.1% (wt/vol) dithiothreitol, 62.5 mM Tris–HCl (pH 6.8), 0.01% (wt/vol) bromophenol blue, and 10% (wt/vol) glycerol, at room temperature for 4–5 h. Then, samples of the above mixtures were placed in boiling water for 5 min and centrifuged for 10 min at 10,000*g*. Ten microliters of supernatant was placed on biphasic polyacrylamide gels (3% stacking gel and 10% separation gel). The electrode buffer was tris-glycine (pH 8.3). Samples were run at 40 mA for 5–6 h. The gels were then stained with Coomassie Brilliant Blue R and G-250 for 24 h, washed with distilled water until the bands were well defined, and photographed under white light. Gels were scored for the presence (1) or absence (0) of every protein band (De la Rosa and González, 2010). These values were then used to compile a data matrix to construct dendrograms based on Jaccard similarity indices and the use of the unweighted pair-group method with arithmetic mean (UPGMA) clustering method.

### Genotypic Data Analysis: Genetic Diversity, Population Structure, and Phylogenetic Analysis

To characterize the different parameters of allelic diversity and genetic variation, the indexes of heterozygosity observed (Ho), heterozygosity expected (He), unbiased expected heterozygosity (uHe), number of different alleles (Na), number of effective alleles (Ne), Shannon’s Information Index (H), fixation index (F), and polymorphic index content (PIC) were calculated using data matrix by fragment size with the GenAlEx6.5 software. Allele frequencies of each cluster, frequency of private alleles, and deviation from Hardy–Weinberg equilibrium (HWE) of loci were also calculated with GenAlEx6.5 (Peakall and Smouse, 2012).

Analysis of molecular variance (AMOVA) method was used for estimating population differentiation directly from molecular markers dataset, based on Dice’s distance, testing a differentiation hypothesis and analyzed by estimating the pairwise fixation index (Fst) using GenAlEx6.5 software (Peakall and Smouse, 2012). AMOVA was used to partition the total collection diversity among and within regions (Spanish and non-Spanish origin) and among and within landraces, commercial cultivars, and wild relatives. The variance components were tested statistically by non-parametric randomization tests using 10,000 permutations, and significance was calculated according to *p* < 0.001.

To estimate the number of hypothetical subpopulations (K) and to quantify the membership probability of each genotype to the inferred subpopulations, Structure V2.3.4 Software was used (Pritchard et al., 2000). The analysis was run for *K* values ranging from 1 to 8 inferred genetic clusters, with 10 independent replicas each, applying a burn-in period of 100,000 steps followed by 100,000 Monte Carlo Markov Chain replicates. Structure Harvester plugin was used to calculate the most adequate *K* value (Earl and VonHoldt, 2012), which implements the Δ*K* method defined by Evanno et al. (2005).

Dissimilarity Analysis and Representation for Windows (DARwin) software was used to determine genetic diversity of the accessions. Clusters analysis was performed to generate a dendrogram using the UPGMA phylogenetic cluster analysis, genetic dissimilarity parameters, and Nei’s unbiased genetic distance or Nei index with DARwin 6.0.15 software (Perrier and Jacquemoud-Collet, 2006).

### Phenotypic Data Analysis

The agromorphological characterization of vetch collection was carried out at “Finca La Canaleja,” Alcalá de Henares, Madrid (602 masl; 40°30′54″ N/03°18′42″ W) during consecutive agricultural seasons 2007–2008 and 2008–2009. The meteorological conditions of this region have an average annual rainfall of 420 mm and an average daily temperature of 13.7°C. The main soil characteristics of this field are as follows: calcium alfisol, loam, moderately alkaline (pH 8.4), organic carbon (0.6%), and saturation of bases (100%). The accessions were grown in 4-m-long, four-row plots with a plot-to-plot spacing of 0.7 m for those placed in the same line and 0.3 m between rows. Four hundred seeds were seeded by accession. A total of 40 morphological and agronomical characters, selected following the recommendation of Bioversity International for species of *Vicia* genus and the UPOV guidelines for *V. sativa* L., were recorded for each plot. Complete and detailed information of the traits studied and the values related to each accession are included in Supplementary Table 8. Mainly, this trait set includes quantitative and qualitative characters, such as size, weight, shape, color related to plant, flower, pod, fruit, seeds, and phenology. Mean values, standard deviation, and maximum and minimum values for quantitative data were calculated using SPSS software. Multivariate statistical analyses of phenotypic data and correlations between different quantitative traits were done with Statgraphics Centurion 19 software, using multiple-variable analysis and PCA Pearson product–moment correlation method.

The characterization of drought-related and yield traits was performed during four agricultural seasons, 2007/2008, 2008/2009, 2016/2017, and 2017/2018, under non-irrigating conditions and sowing conditions as described above. Data on drought-associated parameters [relative water content (RWC), SPAD index, and stomatal conductance (SC)] were evaluated during April and May of the respective years as indicated below, but also relevant traits related to yield, such as seed number, pod number, seed weight, and grain production, together with phenological parameters. Regarding drought response traits, RWC was measured using three plants of each genotype that were analyzed as described (Smart and Bingham, 1974). Leaflets were immediately weighed (W, fresh weight). To estimate the turgid weight (TW), leaflets were incubated in deionized water 24 h at 4°C in dark. Then, plants were oven-dried (80°C) for 2 days to achieve a constant dry weight (DW). Percentage of RWC (%) was calculated as 100 × [(W – DW)/(TW – DW)]. SPAD index was calculated by measuring relative chlorophyll content in a leaf area of 6 mm^2^, using light absorbance in the red (650 nm) and near-infrared (940 nm) with the SPAD-502 meter (Minolta, Japan) as described (Padilla et al., 2018). SC was measured in the field when 50% of plants were flowering on a total of 10 plants per accession and in five leaves per plant on the first expanded leaf using a steady-state leaf porometer (SC-1, Decagon-Devices, LabFerrer, Spain). SC (mmol m^–2^ s^–1^) was done in duplicate and repeatedly during consecutive days as described (De la Rosa et al., 2020). Mean values, standard deviation, and maximum and minimum values for these data were calculated using SPSS software. Statistical analysis of phenotypic data and correlations between different quantitative traits were analyzed by principal coordinate analysis, performed using Statgraphics Centurion software.

### Development of Core Collection

The procedure for the selection of the accessions of the core collection was based on the analysis of the genetic dissimilarity and population structure of the common Spanish vetch CRF collection using SSR molecular markers. Fifty of the total vetch accessions were selected from the different branches of the SSR cluster, using passport^4^ and agromorphological^5^ data as support. To confirm the representativeness of the 50 selected accessions, their location in the cluster of the protein patterns was checked, and it was observed that they were distributed in the different branches of this cluster. After the preliminary selection of the 50 Spanish accessions, a revision of their status of conservation regarding seed availability and germination rate was carried out, and then a total of 47 accessions remained in the VCC. The result of this selection is shown in Table 4, where each entry is identified with its number in the active CRF collection. The evaluation data for agromorphological and drought-associated parameters of the 47 accessions of this VCC are described in Supplementary Table 8.

## Results

### Development of a Standard Set of SSR Reference Alleles for Genotyping and Identification of Accessions of Common Vetch Collection

The availability in the genebank databases of *V. sativa* ESTs has allowed the identification of different polymorphic SSR alleles in coding sequences, although in most cases, the evaluation of polymorphisms and the experimental development and validation of these markers have not been carried out (Chung et al., 2013; Liu et al., 2014; De la Rosa et al., 2020). To analyze their polymorphism and amplification specificity, primers were designed to amplify 24 of these loci. Fourteen of these primers were finally selected because they met the criteria for amplifying a single PCR fragment and presenting polymorphic variants in the samples analyzed (Supplementary Table 3). One of our goals was to develop a minimum set of polymorphic and multiallelic reference markers useful for large-scale genotyping of germplasm collections and the identification of *V. sativa* genotypes. Recently, the combined methodological strategy of multiplex PCR with fluorochrome-labeled primers and subsequent simultaneous analysis of fragments in capillary electrophoresis have allowed the massive genotyping of large numbers of individuals with a minimal set of SSR in high-throughput and cost-effectiveness manner.

A total of 545 common vetch accessions from 12 different countries worldwide (mainly from the Mediterranean basin; Supplementary Table 1), including landraces, cultivars, and wild relatives were analyzed with the 14-SSR primer-pairs set for the identification of polymorphic alleles. All the tested markers were polymorphic for the analyzed accessions. Overall, at least 86 different loci were identified with 2–11 alleles per locus with an average of 6.1 alleles per locus. The SSRs with the highest polymorphism were SSR-138 with 11 polymorphic alleles, followed by SSR-4739S with 10 different alleles. genetic diversity was calculated as polymorphism information content (PIC) ranged from 0.17 to 0.70 with an average of 0.47 ± 0.13, indicating that most of the loci have intermediate or high diversity (Table 1). Total averages of the observed (Ho) and expected heterozygosity (He) were 0.24 and 0.53, respectively, indicating deviation from HWE and high interlocus linkage disequilibrium. Shannon’s Information Index (H) ranged from 0.38 to 1.52 with an average of 0.99 ± 0.08. Other genetic diversity parameters such as Fixation Index (F) ranged from 0.26 to 0.77 with an average of 0.53 ± 0.04 (Table 1). Additionally, although several loci have a low or medium value of gene diversity, and some of them did not seem to show high informative valor, the combined use of data from different markers greatly increases the discrimination capacity of our set of microsatellites. In fact, 99.4% (542 of 545) of the accessions tested with this 14-SSR set have unique and specific profiles, and only three accessions have redundant or duplicate patterns (Supplementary Table 1).

**TABLE 1:** Diversity statistic from 14 SSR tested in accessions of common vetch (*n* = 545; total collection).

Locus	Na	Ne	Ho	He	uHe	F	H	PIC

SSR-310	6	1.98	0.22	0.50	0.50	0.55	1.00	0.46
SSR-102	2	1.88	0.26	0.47	0.47	0.45	0.66	0.36
SSR-179	5	2.12	0.25	0.53	0.53	0.53	0.89	0.44
SSR-184	8	2.58	0.28	0.61	0.61	0.55	1.21	0.55
SSR-073	8	1.86	0.10	0.46	0.46	0.77	0.98	0.44
SSR-140	4	3.00	0.27	0.67	0.67	0.59	1.11	0.59
SSR-217	6	2.68	0.19	0.63	0.63	0.69	1.09	0.55
SSR-129	8	2.23	0.21	0.55	0.55	0.62	1.12	0.50
SSR-138	11	2.73	0.24	0.63	0.63	0.62	1.35	0.60
SSR-115	7	1.90	0.15	0.48	0.48	0.69	0.90	0.42
SSR-439O	4	1.22	0.13	0.18	0.18	0.26	0.38	0.17
SSR-1393P	4	2.40	0.34	0.58	0.58	0.42	1.00	0.52
SSR-4739S	10	3.86	0.49	0.74	0.74	0.33	1.52	0.70
SSR-408Nc	3	1.51	0.20	0.34	0.34	0.41	0.62	0.31

	**Na**	**Ne**	**Ho**	**He**	**uHe**	**F**	**H**	**PIC**
Mean	6.14	2.28	0.24	0.53	0.53	0.53	0.99	0.47
SE	0.71	0.18	0.03	0.04	0.04	0.04	0.08	0.13

### Genetic Diversity, Population Structure, and Cluster Analysis of the Germplasm Collection

**FIGURE 1:**
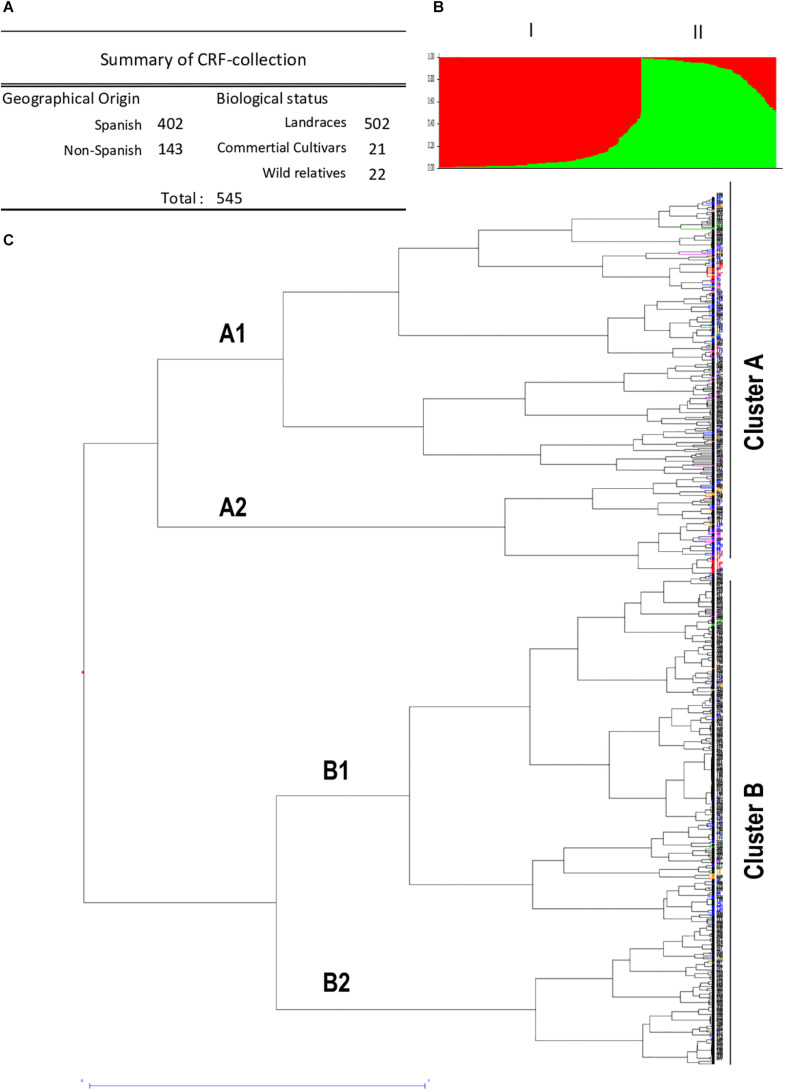
Structure and genetic diversity by geographical origin of common vetch collection. **(A)** Summary of CRF-collection containing 545 *V. sativa* accessions classified by geographical origin and biological status. **(B)** Bar plot showing the genetic diversity structure for the 545 *V. sativa* genotypes based on SSR markers using the program STRUCTURE and *k* = 2 simulation. Different colors represent individuals belonging to clusters A and B. **(C)** Dendrogram generated from 14 SSR primers in 545 common vetch genotypes using hierarchical clustering analysis based on genetic diversity of the tested accessions. Note different geographical origin. Color codes: Spanish (black), Turkish (pink), Greek (blue), Iranian (red), Italian (green), an other non-Spanish (orange) accessions.

The generated SSR dataset matrix of the 545 accessions was used to perform the population structure analysis to determine putative population subdivisions. Bayesian clustering analysis was performed with Structure program to establish clusters of similar genotypes among the different populations. The K analysis provided by the Evanno method (Evanno et al., 2005) did not reveal a clear strong population structure, showing a high probability that the common vetch accessions fell into one unique population. Dissimilarity data were used to construct a hierarchical tree to infer phylogenetic relationships among the 545 *V. sativa*. Jaccard similarity index and UPGMA clustering method (DARWIN software) were used for the analysis of phylogenetic grouping (Figure 1 and Supplementary Figure 1). Unweighted neighbor-joining analysis resulted in a dendrogram with two main clusters or clades with two subclusters for each one. Subpopulations A1, A2, B1, and B2 with 174 (31.9%), 63 (11.6%), 219 (40.2%), and 89 (16.3%) accessions, respectively, can be clearly identified.

Regarding the geographic origin, some accessions from distinct groups showed a slight concordance with their collection sites. However, no clear clustering pattern of geographically close accessions was observed, indicating that the association between genetic similarity and geographical distance was of low significance, which suggests an evolution of the different vetch ecotypes. The analysis of the generated dendrogram indicates a large population dispersion of the Spanish landraces. Almost all accessions from non-Spanish origin are found mainly in cluster A (99/143; 69%) mainly in subcluster A1 (61/143; 43%) (Figure 1). Most of the analyzed populations presented specific and differential SSR patterns, with the most similar populations being the pairs formed by entries 277 and 279, both Andalusian, and 44 and 45, both from Greece. In the case of Iranian varieties, there are two sets of accessions that cluster together, the ones formed by accession numbers 1, 5, and 11, and the ones formed by 2 and 110. The proximity of the values of genetic diversity suggested some genetic redundancy of these genotypes.

The analysis of genetic diversity between 402 Spanish accessions versus the 143 non-Spanish accessions indicates similar mean values of genetic diversity between the two sets of 0.52 and 0.51 in Spanish and non-Spanish accessions, respectively. Other parameters, such as expected heterozygosity (He), Shannon’s information index (H), and PIC, showed similar values in both sets. Only observed heterozygosity (Ho) was significantly lower in non-Spanish ecotypes (Table 2). These results corroborate the diversity of Spanish genotypes and the high degree of similarity observed between the groups, supported by a 0.940 pairwise population matrix of Nei’s value.

**TABLE 2:** Diversity statistic from 14 SSR tested in common vetch accession from Spanish or non-Spanish origin.

Population	N	Na	Ne	Ho	He	uHe	F	PIC	H	Private Alleles
**Non-Spanish**	143	4.500 ± 0.416	2.2302 ± 0.206	0.122 ± 0.018	0.517 ± 0.044	0.519 ± 0.049	0.751 ± 0.039	0.461 ± 0.153	0.946 ± 0.0859	3
**Spanish**	402	2.929 ± 0.699	2.257 ± 0.189	0.280 ± 0.031	0.514 ± 0.042	0.547 ± 0.032	0.442 ± 0.043	0.556 ± 0.175	0.958 ± 0.083	23

The use of AMOVA to estimate the molecular variance among and within-populations showed that most of the genetic variation in common vetch is attributed to within-population variation (95%, Supplementary Table 5). Only 5% of the genetic variation is due to differences among populations of Spanish or non-Spanish origin. The frequency of private alleles (Table 2) also indicates the high diversity of Spanish set versus non-Spanish accessions, supporting that ecotypes of Spanish origin present the most genetic diversity among the common vetch collection.

### Genetic Diversity of Commercial Cultivars and Wild Relatives Versus Landraces of Spanish Origin

Despite the relevance of *V. sativa*, there are few available varieties of commercial cultivars, and CRF collection maintains 21 of these cultivars. Moreover, this collection preserves 21 wild relatives from Spanish origin of great relevance because of their genetic diversity potential. Cultivars are generally believed to have less genetic diversity than landraces. To verify this hypothesis, we carried out a genetic diversity analysis comparing the degree of dissimilarity between landraces versus the commercial cultivars and wild relatives from Spain. Our analysis indicates no drastic differences in genetic diversity between the landraces (mean diversity 0.50), cultivars (mean diversity 0.64), and wild relatives (mean diversity 0.70). As expected, these results indicate that wild taxa have higher genetic diversity than cultivars or landraces. Also, results indicate that cultivars were slightly, but not significantly, more diverse than landraces and corroborate the diversity of wild relatives (Table 3). In fact, the pairwise population matrix of Nei’s unbiased genetic identity or Nei index between the comparisons of these populations ranged from 0.904 to 0.962, indicating a high degree of similarity. AMOVA test showed that only 8% of the genetic variation is due to differences among populations of landraces, wild relatives, and commercial cultivars, indicating that most of the genetic variation is attributed to within-population variation (Supplementary Table 6).

**TABLE 3:** Diversity statistic from 14 SSR tested in Landraces (Ls), wild relatives (WRs), and commercial cultivars (CCs) of Spanish origin accessions.

Population	N	Na	Ne	Ho	He	uHe	F	PIC	H	Private Alleles
**L**	360	5.500 ± 0.653	2.183 ± 0.188	0.294 ± 0.032	0.496 ± 0.043	0.497 ± 0.043	0.393 ± 0.044	0.443 ± 0.149	0.910 ± 0.081	13
**CC**	21	4.000 ± 0.457	2.616 ± 0.264	0.177 ± 0.034	0.559 ± 0.051	0.73 ± 0.052	0.641 ± 0.073	0.504 ± 0.190	1.038 ± 0.115	0
**WR**	21	4.571 ± 0.477	2.933 ± 0.274	0.136 ± 0.025	0.610 ± 0.046	0.625 ± 0.047	0.775 ± 0.033	0.556 ± 0.176	1.160 ± 0.110	6

Landraces and wild relatives of Spanish origin are a great source of genetic diversity. To improve the knowledge of this germplasm, we have carried out an additional characterization of these 381 Spanish accessions using the seed protein profiles to increase the number of molecular markers. A total of 31 protein polymorphic bands distributed in four regions (A, B, C, and D) were observed (Supplementary Table 7), and a great part of these accessions showed different seed protein patterns. The generated matrix of protein bands alleles that scored in terms of presence (1) or absence (0) was analyzed for genetic diversity, and resulting data were used for deeper characterization of these Spanish accessions.

### Vetch Core Collection Genetic Diversity

**FIGURE 2:**
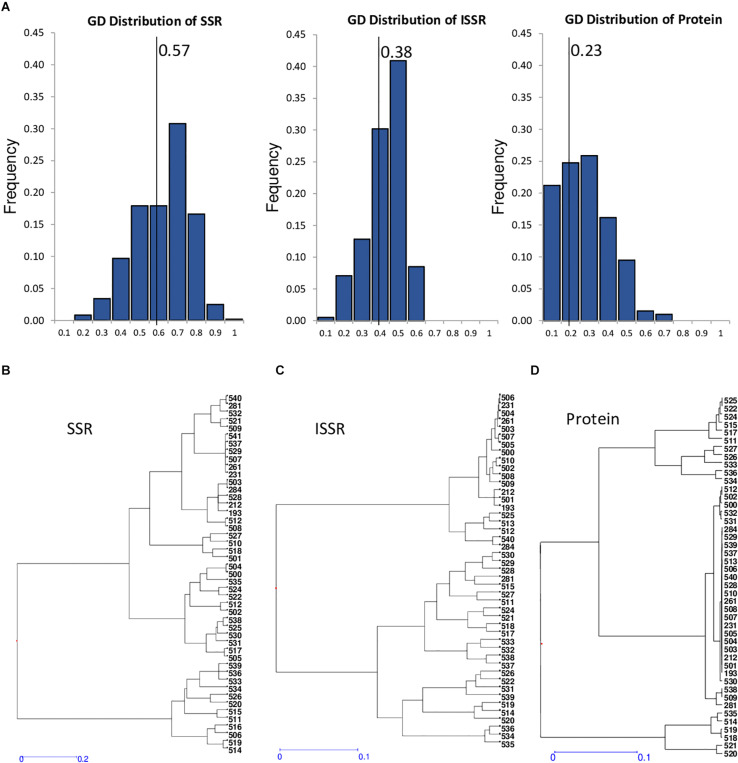
Genetic diversity of core collection with SSRs, seed storage protein profiles, and ISSRs markers. **(A)** Distribution of genetic distances obtained using SSRs, ISSRs, and protein markers for 47 core collection accessions. Solid line indicates mean value of the corresponding genetic distances of the different markers. **(B–D)** Dendrogram generated using hierarchical clustering analysis based on genetic diversity of the core collection accessions using SSRs **(A)**, ISSRs **(B)**, and protein profiles **(C)**.

The analysis of the genetic dissimilarity of the common vetch CRF collection using SSR molecular markers and seed protein patterns allowed the selection of 47 Spanish landraces (Table 4) that constitutes the *V. sativa* core collection (VCC). The representativeness of the 47 selected accessions included in the VCC (12% of total Spanish accessions) was corroborated by analyzing the dispersion and distribution in the different branches of the phylogenetic cluster. The mean genetic diversity value of this core collection is 0.57 (versus 0.50 of total Spanish landraces), supporting the representativeness of the core collections The maximum genetic diversity of the core set by SSR genotyping was detected between genotypes 521 and 514 (GD = 0.93; 92.9%) and the minimum between accessions 503 and 284 (GD = 0.07; 7.1%). Heterogeneous distribution of this set accessions can be observed in the dendrograms of the phylogenetic grouping (Figure 2B). In fact, the pairwise population matrix of Nei’s unbiased genetic identity or Nei index between representative set and the total collection was 0.979, indicating a high degree of similarity.

**TABLE 4:** Core set selected from total common vetch collection (*n* = 47).

Genbank number	Genotype number	Genbank number	Genotype number
BGE001418	500	BGE022205	520
BGE001913	193	BGE022207	521
BGE002035	501	BGE022210	281
BGE004285	212	BGE022211	522
BGE004317	503	BGE022247	524
BGE004324	504	BGE024712	525
BGE004334	505	BGE024720	526
BGE004352	231	BGE025285	528
BGE004375	506	BGE025287	529
BGE004394	507	BGE025611	530
BGE004409	508	BGE026276	531
BGE004422	261	BGE026420	527
BGE005449	502	BGE029065	532
BGE007271	509	BGE029691	533
BGE014897	510	BGE029927	534
BGE014900	511	BGE029949	535
BGE014901	512	BGE029959	536
BGE014902	513	BGE035400	537
BGE016911	517	BGE036257	538
BGE016914	518	BGE036258	539
BGE016923	516	BGE037817	284
BGE016970	515	BGE040344	540
BGE017178	514	BGE040346	541
BGE019772	519		

*Genotype numbers are shown in clustering trees for simplicity.*

To obtain a more accurate genetic characterization of the VCC, the accessions were further characterized using ISSR genetic molecular markers (Supplementary Table 4). A total of 129 polymorphic fragments have been identified from 221 to 3,490 bp. The ISSR 8 showed the highest polymorphism with 19 alleles. ISSR 20 had the lowest polymorphism with eight alleles. Using these data, a present/absence matrix was constructed to analyze the results. Genetic distance between accessions was calculated using Nei Index, included in NTSYS software (Rolf, 1998), and the UPGMA method was used to cluster accessions. All the genotypes showed different ISSR patterns, being the most similar 231, 506, 504, and 261, all of them collected in Andalucía (South of Spain). The accession 526, a weedy population collected in Asturias (North of Spain), and 539, collected in Malaga province, are the most separated materials (Figure 2C). Analyses of distribution of genetic distances comparing these three different markers, SSR, ISSR, and protein profiles, have mean values of 0.57, 0.38, and 0.23, respectively (Figure 2A). This comparative analysis indicates that SSR is the most genetically informative marker. In fact, ISSR and protein profiles have lower diversity, heterozygosity, and polymorphism indexes (Table 5). Dendrograms generated by genetic distances values using unweighted neighbor-joining analysis with DARWIN software show different phylogenetic clustering, suggesting that different markers have different informative content (Figures 2B–D). Structure analysis of this VCC with the three markers did not support the existence of a clear strong population structure, showing a high probability that the common vetch accessions of the collection belong to a unique population.

**TABLE 5:** Diversity statistic from SSRs, ISSRs, and proteins tested in core collection of common vetch (*n* = 47 accessions).

	SSR	Protein	ISSR
N loci	68	35	129
Na	4.857 ± 0.523	1.857 ± 0.083	1.984 ± 0.016
Ne	2.569 ± 0.204	1.455 ± 0.058	1.533 ± 0.029
He	0.569 ± 0.043	0.422 ± 0.038	0.316 ± 0.013
uHe	0.575 ± 0.043	0.275 ± 0.029	0.320 ± 0.013
H	1.072 ± 0.097	0.278 ± 0.029	0.480 ± 0.017

### Evaluation of the VCC for Phenotypic Traits

The agromorphological characterization of the vetch collection was done during two consecutive agricultural seasons, as detailed above. This evaluation includes measures of relevant quantitative and qualitative traits shown in Supplementary Table 8. A summary of the quantitative studies, which includes phenological data, whole-plant characterization, and different measures of size, weight, shape or color related to plant, flower, pod, fruit, and seeds, is shown in Table 6A. The analysis of correlations between these quantitative traits by multiple-variable analysis and PCA Pearson product–moment correlation methods does not show statistically relevant correlations. Slight, although statistically significant, inverse correlations are observed between phenology and seed size and seed weight, suggesting a small negative impact between early flowering and production.

**TABLE 6:** Measurements of agromorphological traits **(A)** and drought-associated parameters **(B)** in accessions of the core collection.

A				
	Quantitative agromorphological traits *			
	Average	SD	Max	Min
**Flowering**				
Days to first flowering (d)	157.8	11.4	186.0	144.0
Days to 50% flowering (d)	170.0	12.1	207.0	153.0
Days to maturity (d)	222.5	4.7	233.5	214.0
**Plant**				
Height (cm)	74.8	16.7	107.9	44.0
Diameter (cm)	28.8	8.6	47.8	12.9
No. of primary branches	2.5	0.6	4.8	1.8
**Leaf**				
Leaf length (cm)	6.7	1.4	8.6	3.6
No. leaflets by leaf	12.2	1.5	14.4	8.0
Leaflet length (cm)	2.0	0.3	3.0	1.4
Leaflet width (cm)	0.8	0.2	1.4	0.4
**Flower**				
No. of flowers by peduncle	1.4	0.3	2.3	1.0
Mean length standard petal (cm)	2.3	0.3	2.7	1.5
Mean width the standard petal (cm)	1.7	0.2	2.1	0.9
Claw standard petal length (cm)	0.9	0.2	1.7	0.5
Claw standard petal width (cm)	0.6	0.1	0.7	0.4
Calyx length (cm)	1.3	0.2	1.6	0.7
Calyx teeth length (cm)	0.7	0.1	0.9	0.3
**Pod/seed**				
Pod length (cm)	5.3	0.7	6.2	3.5
Pod width (cm)	0.4	0.1	0.6	0.2
Pod thickness (cm)	0.4	0.1	0.4	0.2
Seeds per pod	8.1	0.9	10.9	7.1
Seeds bad developed per pod	1.2	0.4	2.3	0.4
Seed length (cm)	0.5	0.1	0.6	0.2
100 seed weight (g)	5.2	2.2	10.2	0.9

**B**				

	**Quantitative drought-related and yield traits**			
	**Average**	**SD**	**Max**	**Min**

**Drought-related parameters**				
SC (mmol × m^–2^ × sg^–2^)	189.8	86	482	30.2
RWC (%)	90.6	5.1	102.6	69.4
SPAD index	43.6	4.1	51.4	35.2
**Yield related parameters**				
Seed num/pod	6.4	1.1	9.3	2.9
Pod num/plant	48.5	38	167.3	5.5
Seed num/plant	322.7	283.7	1421.6	21.5
Weight 100 seeds (g)	6.6	2.9	12	0.9
Grain weight/plant (g)	17.7	13.7	67.1	0.7
**Phenology**				
Flowering day (days to 50% flow)	156.6	9.8	199	147

*Detailed data and descriptors are shown in Supplementary Table 8.

**FIGURE 3:**
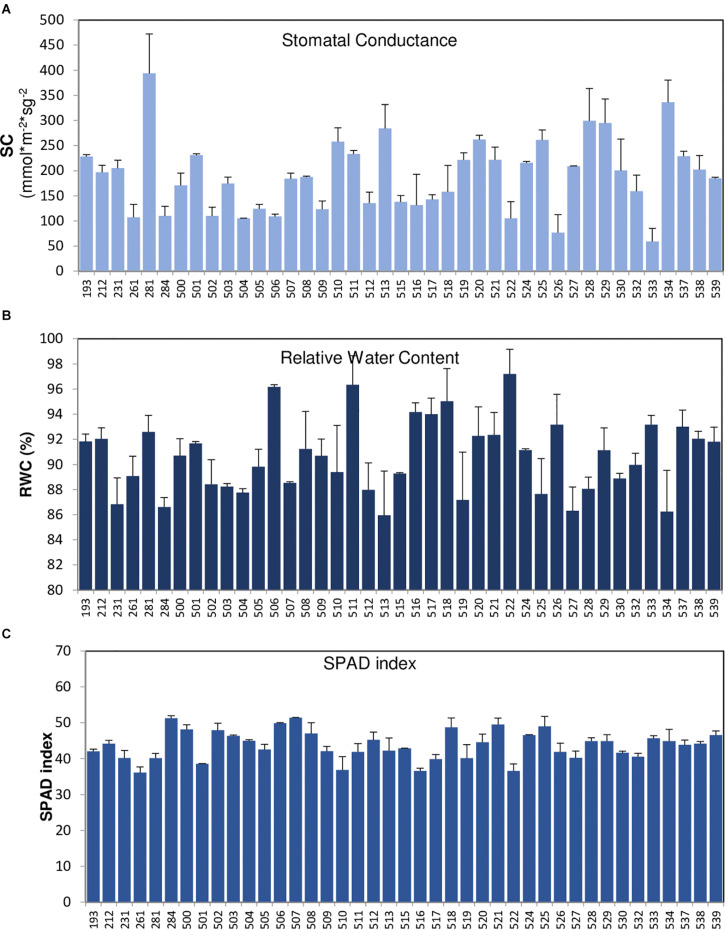
Measurements of drought-associated parameters measured in accessions of the core collection. Stomatal conductance **(A)**, relative water content **(B)**, and SPAD index **(C)** of the core collection (VCC) accessions were measured in non-irrigation conditions, on a total of 10 plants per accession. The error bars indicate standard deviation.

In order to identify new sources of drought tolerance varieties, the VCC was also phenotyped for several agronomic traits, including yield and drought-associated parameters, as SC, SPAD index, or RWC (Figures 3A–C). Table 6B shows the results for the VCC evaluation of drought tolerance parameters and yield measures, as seed number, pod number, seed weight, and grain production together with phenological parameter (days to flowering). The parameter in which we have found the greatest variations was SC (Figure 3A). Recently, this parameter has been shown to be crucial to allow the identification of potential drought-tolerant and drought-sensitive common vetch varieties (De la Rosa et al., 2020), supporting the use of this parameter to identify *Vicia* varieties potentially tolerant to water stress. Based on this characterization approach, we can identify interesting breeding candidates from this VCC, such as accessions 281, 528, 529, or 534 with good yield levels and optimal values of SC. All this information can be used in the future in improvement programs or in the direct use of these common vetch varieties.

### Validation of *V. sativa* SSR Set to Others *Vicia* Species

**FIGURE 4:**
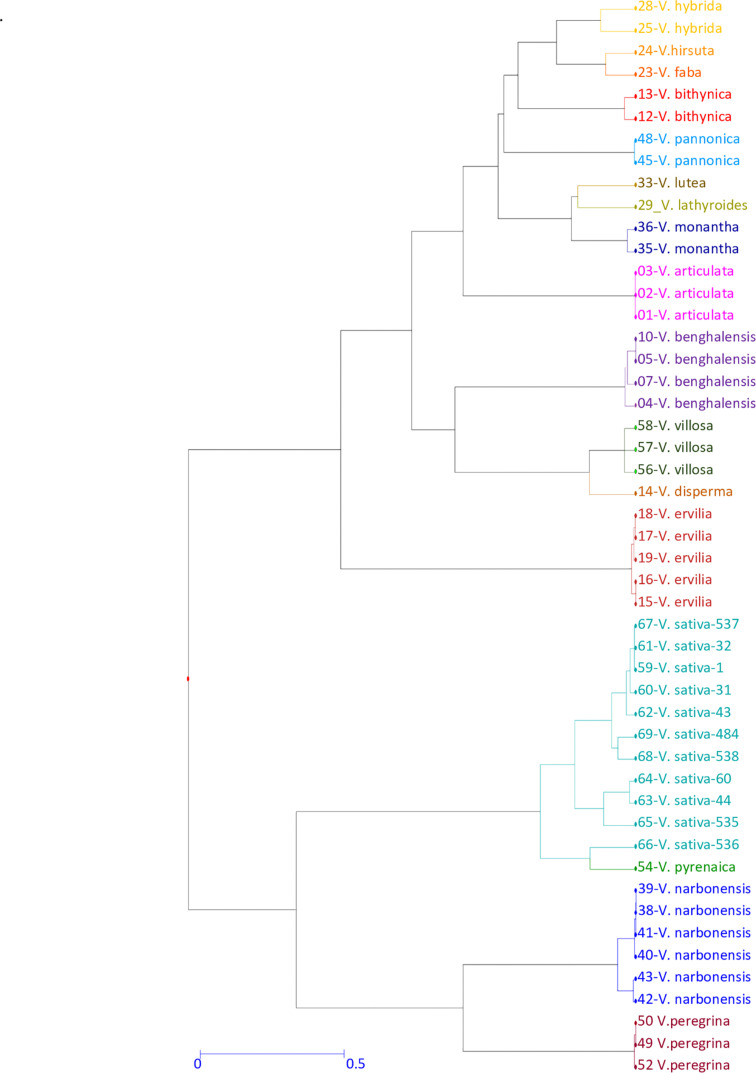
Genetic diversity of the *Vicia* genus genotypes. Dendrogram generated from 9 SSR primers across 38 accessions from 16 different *Vicia* species. Different colors represent different *Vicia* species.

To analyze the potential cross-amplification ability of our *V. sativa* EST-SSR set across another 16 *Vicia* species, we selected 38 accessions from different species of *Vic*ia genus (Supplementary Table 2) plus 11 *V. sativa* accessions as control. Marker SSR-184 from  *V. sativa*   did   not   amplify any   fragment reproducibly and was discarded for further analysis. The number of alleles (N_*A*_) per locus of the remaining nine SSRs varied widely among them, with values ranging from 13 to 22 with an average of 17.8 alleles per locus. The most polymorphic allele was SSR-138 with 22 fragments. Total averages of the observed (Ho) and expected heterozygosity (He) were 0.15 and 0.89, respectively, indicating high deviation from HWE and high interlocus linkage disequilibrium, as expected because different species were analyzed. Genetic diversity was calculated as PIC ranged from 0.82 to 0.92 with an average of 0.88 ± 0.04, indicating that most of the loci have very high diversity. Shannon’s Information Index (H) ranged from 2.08 to 2.77 with an average of 2.47 ± 0.08. Other genetic diversity parameters as Fixation Index (F) ranged from 0.61 to 0.93 with an average of 0.83 ± 0.03 (Table 7). These enormous discrepancies between Ho and He and high values of the diversity index have been previously described in similar works (Raveendar et al., 2015). To analyze phylogenetic clustering, the generated diversity matrix was used for unweighted neighbor-joining analysis with DARWIN program to generate a dendrogram (Figure 4). In this tree, *Vicia pyrenaica* and *Vicia sativa* appear in the same clade, as *Vicia narbonensis* and *Vicia peregrina* seem to be closely related in the same clade. *Vicia disperma, Vicia benghalensis*, and *Vicia villosa* exhibit great similarity to each other.

**TABLE 7:** Diversity statistic from the nine SSRs tested in accession of different species of *Vicia* genus.

Locus	Na	Ne	Ho	He	uHe	H	F	PIC
SSR-310	14	9.08	0.18	0.89	0.90	2.40	0.79	0.88
SSR-102	13	6.06	0.16	0.83	0.84	2.08	0.80	0.82
SSR-179	17	5.97	0.33	0.83	0.84	2.22	0.61	0.82
SSR-073	20	10.91	0.12	0.91	0.92	2.61	0.87	0.90
SSR-140	20	9.94	0.08	0.90	0.91	2.61	0.91	0.89
SSR-217	21	12.17	0.06	0.92	0.93	2.73	0.93	0.91
SSR-129	19	9.92	0.14	0.90	0.91	2.55	0.84	0.89
SSR-138	22	12.64	0.16	0.92	0.93	2.77	0.82	0.92
SSR-115	14	7.66	0.10	0.87	0.88	2.25	0.88	0.86

	**Na**	**Ne**	**Ho**	**He**	**uHe**	**H**	**F**	**PIC**

Mean	17.78	9.37	0.15	0.89	0.89	2.47	0.83	0.88
SE	1.128	0.809	0.026	0.011	0.011	0.081	0.032	0.035

## Discussion

Genetic diversity from locally adapted landraces is essential to develop future crop productivity. Two of the major challenges of plant genetic resource collections worldwide are the elimination of redundant duplications within and between different genebanks and the traceability of accession identity. Traditionally, the identification of genebank accessions has been based on passport records from taxonomic and place of collection data and the characterization of morphoagronomic traits. The enlargement of these traditional descriptors with novel molecular marker data can help to improve the management of seed collections (Mascher et al., 2019). Our interest was focused on the design and validation of new molecular tools for the characterization of vetch PGR. Recent efforts in transcriptome analysis using NGS technology provide a relatively cost-effective and useful source for molecular markers in unsequenced species, such as vetch. These technologies have allowed the sequencing of ESTs and the identification of polymorphic variants that facilitate the design of molecular markers, such as SSR or SNPs. In the current scenario, our work was aimed at the characterization of the 545 accessions of Spanish common vetch collection with a minimal standard set of 14 SSRs markers to study the genetic diversity and population structure of this collection. The combined use of simultaneous multiplex PCR techniques with fluorochrome-marked primers and the use of automatic sequencers allowed the genotyping of hundreds of individuals with a set of high-yield and cost-effective markers. Similar approaches have been previously done in tomato, grapevine, and wheat (Bredemeijer et al., 2002; Röder et al., 2002; This et al., 2004). The selected set of SSR molecular markers provides enough polymorphic variants combinations to analyze the genetic diversity of the common vetch. Furthermore, more than 99.4% of the total analyzed varieties have a unique and specific microsatellite profile, allowing the identification of duplicates and closely related varieties, suggesting its potential value and future use in varietal identification.

The taxonomy of the *Vicia* genus is complex, and the exclusive use of agromorphological traits, in some cases, is not sufficient to clarify the genetic relationships between the species of this genus (Haider and El-Shanshoury, 2000). In recent years, an effort has been made to design and use transferable markers to different legume species (Akash and Myers, 2012; Jafari et al., 2013), and emphasis has been done in the cross-species transferability of markers from the *V*. *sativa to* other distantly related species of the *Vicia* genus. The high level of cross-species transferability observed in the study of Raveendar’s laboratory (Raveendar et al., 2015) indicates that the *V*. *sativa* cDNA-derived *SSR* may be suitable for analysis of genetic diversity and population structure, construction of high-density linkage maps, conservation, and molecular marker-assisted breeding of target species. In our case, the fact that these SSRs are located in coding regions of the genome, less polymorphic but potentially more conserved than non-coding regions, probably has favored the high rate of transferability across species, as previously suggested (Scott et al., 2000; Raveendar et al., 2015). In our work, 9 of these 14 SSR markers have shown a high degree of cross-transferability to 16 species of *Vicia* genus: *articulata*, *benghalensis*, *bithynica*, *disperma*, *ervilia*, *faba*, *hirsuta*, *hybrida*, *lathyroides*, *lutea*, *monantha*, *narbonensis*, *pannonica*, *peregrina*, *pyreneica*, and *villosa*. Different taxonomic methodologies and phylogenetic analyses based on the use of molecular markers and genomic size estimations, including repeated genomic sequences, have been used to characterize the GD of the genus *Vicia* (Sanz et al., 2007; Macas et al., 2015). Our results agree with these previous studies and indicate a high level of transferability of SSRs across *V. sativa* and related species of *Vicia* genus. Additionally, these analyses support the potential use of the SSR set in genetic diversity and phylogenetic studies for other *Vicia* species.

The *V. sativa* aggregate is a complex and dynamic group, including cultivated, weed, and wild forms. Members included in this aggregate have great variability of geographical origin, ecology, karyotype, and morphology. This fact makes the classification of its members difficult, and historically, different taxonomies have been proposed to organize them. Previously, some authors have recognized that this aggregate contains related species, whereas other authors consider these taxa as different subspecies or varieties within the *sativa* species. One of the more recent taxonomic classifications based on molecular data proposed that the *V. sativa* aggregate has six subspecies: subsp. *nigra* (L.) Ehrh.; subsp. *segetalis* (Thuill.) Gaudin; subsp. *cordata* (Wulfen ex Hoppe); subsp. *amphicarpa* (L.) Batt.; subsp. *sativa*; and subsp. *macrocarpa* (Moris) (van de Wouw et al., 2001). This complex taxonomic situation has been reviewed by De la Rosa and González (2010). Our study includes accessions of *nigra, cordata*, *amphicarpa*, and *macrocarpa*. The cluster obtained shows that the accessions of the same taxa are not grouped, but the different accessions of the different taxa are located throughout the dendrogram. This result reinforces the idea that the different taxa of the sativa aggregate are subspecies or varieties instead of different species.

Focusing on the *V. sativa* collection, the phylogenetic analysis of dendrograms suggests that accessions can be divided into two different clusters, although some aggregations at the level of country provenance were observed. For instance, most of the Mongolian varieties showed clustering with each other, and individuals originating from Iran also grouped together. In contrast, Greek accessions did not cluster tightly, neither fall into the same main cluster, and the same observation applies to Turkish accessions. Most of the Spanish accessions along with some Greek and Turkish samples, despite their different geographical origin, grouped together, indicating that they could have originated in the same place and that their diversification has not been too strong. These results are according to the archeological evidence that suggests that the Mediterranean region is the principal center of diversification of the common vetch (Maxted and Bennett, 2001; Bouby and Léa, 2006; de Vareilles et al., 2020). We have not observed great differences between Spanish and non-Spanish genotypes when genetic diversity is analyzed, and the great dispersion of Spanish accessions along the dendrogram could indicate that Spain is a potential center of diversification of this species. Our analysis did not find significant differences in genetic diversity between landraces and cultivars, suggesting that the genetic variability of commercial cultivars has not been lost because of the breeding process or that cultivars are directly selected landraces.

As previously discussed, one of the problems in genebanks is the large number of samples in some collections, with the difficulty of maintenance, characterization, and documentation and consequently their utilization. This problem could be solved by reducing the number of accessions, establishing core collections that include a minimum number of non-redundant individuals containing the maximum variability of the entire germplasm collection and avoiding redundancy that does not contribute to the diversity of the collection. The core collection characterization and evaluation are easier and faster than the analysis of an entire germplasm collection. In the present work, SSR molecular markers have been used to elucidate the genetic diversity of the vetch collection. The analysis of genetic variability and population structure, together with passport information and morphological data, has allowed the selection of 47 accessions, to create the Spanish common VCC, which would constitute the primary resource for a genetic breeding program with minimum loss of genetic diversity of this important genetic resource. These selected 47 individuals represented more than 80% of total accession diversity and showed a higher heterozygosity and polymorphic average values (He = 0.57 ± 0.04, PIC = 0.56 ± 0.08) than those of the original collection (He = 0.53 ± 0.04; PIC = 0.47 ± 0.13), despite constituting approximately 10% of the latter, suggesting that the VCC maintains most of the genetic diversity of the whole large collection. These diversity studies have been complemented with other marker analyses as seed storage protein patterns and ISSR. The estimation of genetic diversity was higher using SSR, followed by ISSR and protein profiles. Despite the differences among marker types, the three markers support that germplasm collection lacks a clear structure linked to their geographical origin, but it is possible to differentiate all the accessions. The inherent differences or discrepancies between results should be attributable to the three kinds of markers targeting different genome sequences that can be or not coding regions or participate in the gene expression or the association methods used to calculate genetic diversity. Dissimilarity calculations from allelic data from SSR used Simple Matching index, whereas single data of presence/absence data matrix use the Dice method. In these analyses, the SSR markers are those with the highest degree of polymorphism, genetic diversity, and consistency due to their multiallelic nature. Our results are similar to those obtained in diversity studies of other germplasm collections, which also have used different molecular markers, indicating the difficulty to compare results between markers showing different levels of polymorphism (Liu et al., 2018; Mahmoud and Abd El-Fatah, 2020).

The results of this large-scale molecular characterization are essential for the management and conservation of the common vetch collection, as well as providing information and guidance for its rational use by the research community and the legume companies in their breeding efforts. Our results provide theoretical support for the analysis of genetic diversity structure and evolution of the international common vetch collection, necessary to develop new approaches for collecting, conserving, and using germplasm resources. Furthermore, the genomic information provided by this study can facilitate marker-assisted selection for vetch improvement programs. Also, the VCC was phenotyped for several agronomic traits to evaluate its genetic and phenotypic diversity and identify new sources of drought tolerance and high yield varieties. Previously, it has been shown that the measurement of different parameters to identify drought-tolerant and drought-sensitive genotypes seems to depend on the species under study, as different species may possess different mechanisms of drought tolerance (Silva et al., 2011). In the present work, we use the SC, RWC, and SPAD index that have been shown to correlate with drought tolerance in different species, including common vetch (Colom and Vazzana, 2003; Li et al., 2006; Silva et al., 2011; De la Rosa et al., 2020). The SC is the parameter with the greatest variation. Recently, this parameter has been shown to be crucial to the identification of potential drought-tolerant and drought-sensitive common vetch varieties (De la Rosa et al., 2020), supporting the use of this parameter to identify *Vicia* varieties potentially tolerant to water stress. Based on this characterization approach, we can identify interesting breeding candidates from this VCC, such as accessions 281, 528, 529, or 534 with good yield levels and optimal values of SC. This information can be used in the future in improvement programs or the direct use of these common vetch varieties. All these materials are potential sources of resistance to drought abiotic stress of undoubted interest in the actual climate change scenario.

## Conclusion

The results of this study provided a detailed genetic analysis of the common vetch germplasm collection maintained by the CRF-INIA, which includes accessions collected throughout the world. We have developed a set of 14 SSRs that have allowed the characterization of a collection of 545 accessions of *V. sativa*. We have demonstrated that these markers are transferable to other species of the genus *Vicia*. Using passport, agromorphological, seed storage proteins, SSR, and ISSR data, we have constructed a VCC of Spanish origin including 47 accessions. This core collection comprises genetically and phenotypically diverse germplasm, including traits related to yield and drought tolerance, ready to be used in vetch breeding programs.

## Data Availability Statement

The original contributions presented in the study are included in the article/Supplementary Material, further inquiries can be directed to the corresponding author.

## Author Contributions

LD, JG, and ER-P conceived and designed the experiments. LD, EZ, and TM-P performed and analyzed phenotypic data. LD, JG, ML-R, and ER-P performed and analyzed genotypic data. LD and ER-P wrote the manuscript. JG, ML-R, EZ, and TM-P provided critical review of the manuscript. All authors read and approved the final manuscript.

## Conflict of Interest

The authors declare that the research was conducted in the absence of any commercial or financial relationships that could be construed as a potential conflict of interest.

## Footnotes

1 www.fao.org/faostat

2 https://www.genesys-pgr.org/

3 https://eurisco.ipk-gatersleben.de/

4 https://bancocrf.inia.es/en/

5 http://webx.inia.es/web_coleccionescrf/CaracterizacionCRFeng.asp

## Supplementary Material

Supplementary Figure 1 Genetic diversity of common vetch genotypes and type of accessions. Dendrogram generated from 14 SSR primers in 545 common vetch genotypes using hierarchical clustering analysis based on GD of the tested accessions. Color codes: Spanish landraces (black), non-Spanish landraces (blue), commercial cultivars (red), and *wild relatives* (green) accessions.

Supplementary Table 1 Accession numbers of the common vetch collection analyzed in this work and data matrix of fragment size by 14-SSR genotyping.

Supplementary Table 2 Accessions of different species of *Vicia* genus analyzed in this work- and data matrix of fragment size by nine-SSR genotyping.

Supplementary Table 3 Gene-specific primers used for Multiplex PCR amplification.

Supplementary Table 4 ISSR primers.

Supplementary Table 5 Analysis of molecular variance (AMOVA) using 14 SSR data tested in accession from Spanish or non-Spanish origin.

Supplementary Table 6 Analysis of molecular variance (AMOVA) using 14 SSR data tested in landraces, wild relatives.

Supplementary Table 7 Diversity statistic from protein profiles tested in accession of common vetch (*n* = 381 accessions/31 loci).

Supplementary Table 8 Descriptors for *Vicia sativa* L., and phenotypic characterization data of the VCC accessions.
