# Supplementary material for: Common Vetch, Valuable Germplasm for Resilient Agriculture: Genetic Characterization and Spanish Core Collection Development
Source: Front Plant Sci. 2021 Mar 9;12:617873. doi: 10.3389/fpls.2021.617873 (PMC7985455; doi:10.3389/fpls.2021.617873)
Supplement: Supplementary Figure 1 — Genetic diversity of common vetch genotypes and type of accessions. Dendrogram generated from 14 SSR primers in 545 common vetch genotypes using hierarchical clustering analysis based on GD of the tested accessions. Color codes: Spanish landraces (black), non-Spanish landraces (blue), commercial cultivars (red), and wild relatives (green) accessions. [file Presentation_1.PPTX]

## Slide 1
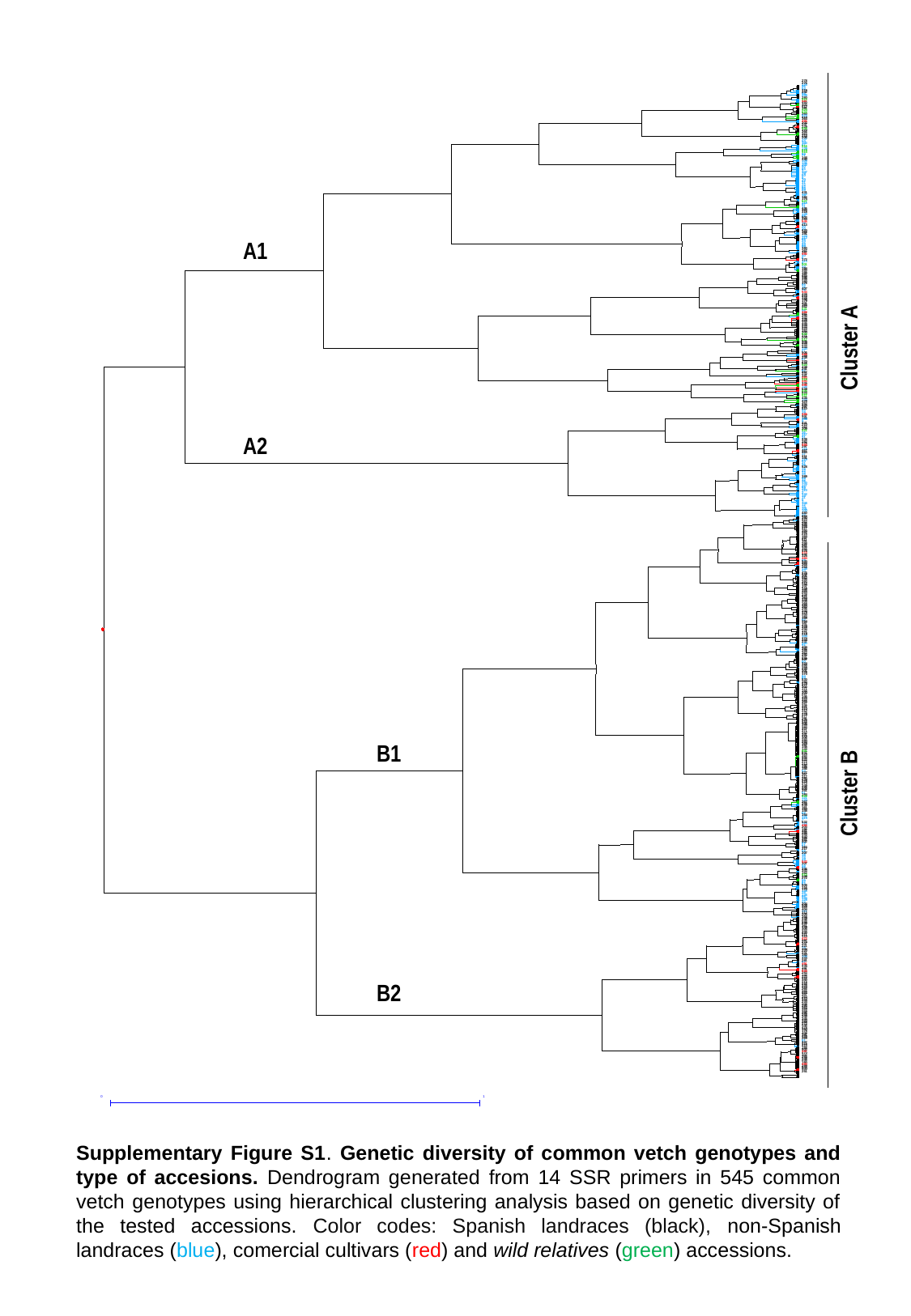

278
275
280
260
284
271
270
282
276
297
262
256
252
257
298
261
268
264
254
292
288
259
294
279
277
291
290
250
293
267
286
285
249
272
287
274
281
283
266
265
253
295
263
296
258
289
273
269
255
251
125
137
103
139
144
109
106
102
107
122
118
129
124
112
99
113
134
126
123
117
116
141
119
130
127
110
132
108
101
100
138
104
140
146
148
143
121
120
145
131
114
115
135
136
133
128
111
105
147
142
96
54
98
50
92
77
90
85
79
60
70
72
52
94
89
97
71
88
76
66
95
75
56
67
62
93
64
78
73
49
65
82
91
80
84
83
53
51
68
69
61
58
59
55
87
63
86
74
57
81
319
318
342
338
335
309
311
325
328
308
344
329
337
336
324
301
303
300
323
322
327
326
321
310
313
341
315
306
317
307
346
343
316
312
330
339
305
332
314
347
304
333
331
340
334
348
345
320
302
299
242
202
247
229
214
206
240
200
209
233
238
241
232
210
215
201
212
199
228
243
219
244
237
230
227
213
226
203
217
207
204
205
239
236
235
211
221
220
231
223
222
218
216
225
224
234
208
246
248
245
499
512
524
522
523
535
516
519
514
527
503
545
501
543
531
534
517
505
506
526
520
518
539
536
533
515
511
510
521
528
540
507
530
541
537
529
525
538
532
542
513
508
509
544
502
504
500
487
450
495
492
454
474
462
488
461
468
489
449
457
481
483
493
496
452
485
498
494
467
464
480
453
466
458
456
455
475
473
484
460
476
463
471
479
477
482
486
451
469
472
491
465
478
459
497
470
490
403
412
414
431
446
413
420
407
428
401
445
434
433
424
409
444
435
429
443
447
441
438
423
417
411
442
448
421
425
400
426
416
406
399
432
422
427
439
419
405
440
402
404
430
436
410
408
437
418
415
181
182
174
165
191
167
172
194
193
195
197
180
176
198
179
166
189
186
162
153
151
149
164
168
183
159
187
177
173
171
155
175
169
157
192
185
156
184
158
161
160
154
196
163
152
170
150
178
188
190
364
370
366
363
353
380
387
396
398
376
386
383
397
389
390
394
368
395
393
355
384
360
356
379
354
375
374
392
388
362
378
365
382
377
371
352
372
367
373
349
351
381
385
358
361
357
369
359
350
391
47
3
8
42
31
39
30
20
24
45
44
40
6
4
34
16
27
29
9
36
37
21
17
41
25
22
35
32
26
28
2
14
11
5
1
12
10
23
48
43
7
18
15
19
13
46
38
33
0
1
A1
Cluster A
A2
B1
Cluster B
B2
Supplementary Figure S1. Genetic diversity of common vetch genotypes and type of accesions. Dendrogram generated from 14 SSR primers in 545 common vetch genotypes using hierarchical clustering analysis based on genetic diversity of the tested accessions. Color codes: Spanish landraces (black), non-Spanish landraces (blue), comercial cultivars (red) and wild relatives (green) accessions.
