# Supplementary material for: Common Vetch, Valuable Germplasm for Resilient Agriculture: Genetic Characterization and Spanish Core Collection Development
Source: Front Plant Sci. 2021 Mar 9;12:617873. doi: 10.3389/fpls.2021.617873 (PMC7985455; doi:10.3389/fpls.2021.617873)
Supplement: Supplementary Table 4 — ISSR primers. [file Table_1.DOCX]

Supplementary Material

# Supplementary table S1: Accession numbers of the common vetch collection analyzed in this work and data matrix of fragment size by 14 SSR genotyping. See Data sheet table 1

# Supplementary table S2: Accessions of different species of Vicia genus analyzed in this work- and data matrix of fragment size by 9 SSR genotyping. See Data sheet table 2. Supplementary table S3: Gene-specific primers used for Multiplex PCR amplification

| ***Name*** | ***Forward**** | ***Reverse*** | ***Fluorescent Probe*** | ***SSR*** |
| --- | --- | --- | --- | --- |
|  |  |  |  |  |
| Vs-SSR-310 | GGGTGCCCTAGCATTTGT | ATCTCCGGCGTCAGTTTC | HEX | (CTC)_6_ |
| Vs-SSR-102 | TTCAACGGAGATGGATCG | CGTCTTCTTTCAGAGGCG | HEX | (GTT)_5_ |
| Vs-SSR-179 | AGCTATGCGAGAGGCTCC | CTGTGGGAAGGCACATCT | HEX | (TGA)_6_ |
| Vs-SSR-185 | CTCCTCAATTTTCCCCCA | TCCTCTGCAGCTTCACTTCA | HEX | (CAT)_5_ |
| Vs-SSR-073 | CCTCCCAATCCTCCATTC | CCCTAGTCCTCCAATTTCG | 6-FAM | (GTT)_5_ |
| Vs-SSR-140 | TTGCTTTGATGTTTGGAGC | CCCTAAATTCCCAACCCA | 6-FAM | (GGT)_7_ |
| Vs-SSR-217 | CCATCGCCACCACCAAAC | ACAAAGAAATTGTTCTACTATCTGCAT | 6-FAM | (AAC)_7_ |
| Vs-SSR-129 | AGGAGAGGCAAGGACCAG | CTTTTTCTCTAACTCATTCATGTC | PET | (GA)_10_ |
| Vs-SSR-138 | CACTGTGACTCAGTTTCGTTG | CGATTTTGAACCCTAACCG | NED | (TTA)_7_ |
| Vs-SSR-115 | CATAAACAAGGGCAAGAAAA | GAGGAAAACATTGGTGGGA | NED | (TGC)_6_ |
| Vs-SSR-439O | CCCCATGGCCTTCTCCTTAC | AGTTCTTCACTCACACTTCATCA | 6-FAM | (GGTAGT)_3_ |
| Vs-SSR-1393P | TCCACCTTTCCACTCATGCT | TGCTGCATCATGGACAAGACT | HEX | (CT)_6_ |
| Vs-SSR-4739S | ATCCTTAGCCAGTTTCTTCGCA | CCGAAGGCGATAAACTCTCCA | HEX | (ATG)_7_ |
| Vs-SSR-408Nc | TCTTCTCCAAAGCCTTCACTTGA | TTGGCGTTGTTTAAGATACCC | 6-FAM | (GTT)_6_ |
|  |  |  |  |  |
| * Forward oligonucleotides were marked with the indicated fluorescent dye | | | |  |

**Supplementary table S4:** ISSR primers

| Primer | Sequence 5' -> 3' |
| --- | --- |
| **ISSR 8** | AGAGAGAGAGAGAGAGYC |
| **ISSR 9** | GAGAGAGAGAGAGAGAA |
| **ISSR 11** | GAGAGAGAGAGAGAGAYG |
| **ISSR 12** | ACACACACACACACACYT |
| **ISSR 14** | CACACACACACAGT |
| **ISSR 15** | CACACACACACAAG |
| **ISSR 17** | GTGGTGGTGGC |
| **ISSR 19** | CACACACACACACACARC |
| **ISSR 20** | ATGATGATGATGATGATG |
| **ISSR 21** | AGAAGAAGAGAGAGAGYT |
|  | Y=C,T;  R=A,G |

**Supplementary table S5:** Analysis of Molecular Variance (AMOVA) using 14 SSR data tested in accession from Spanish or non-Spanish origin

| **Source** | **df** | **SS** | **MS** | **Estimated Variation** | **% Variation** | **p value** |
| --- | --- | --- | --- | --- | --- | --- |
| **Among Pops** | 1 | 86.603 | 86.603 | 0.197 | 5% | <0.001 |
| **Within Pops** | 1088 | 3927.913 | 3.610 | 3.610 | 95% | <0.001 |
| **Total** | 1089 | 4014.517 |  | 3.807 | 100% | <0.001 |
|  |  |  |  |  |  |  |
| **df** degree of freedom , **SS** Sum of Squares, **MS** Mean of Squares | | | | |  |  |

**Supplementary table S6:** Analysis of Molecular Variance (AMOVA) using 14 SSR data tested in landraces, wild relatives

| **Source** | **df** | **SS** | **MS** | **Estimated Variation** | **% Variation** | **p value** |
| --- | --- | --- | --- | --- | --- | --- |
| **Among Pops** | 2 | 47.821 | 23.910 | 0.263 | 8% | <0.001 |
| **Within Pops** | 801 | 2845.428 | 3.552 | 3.552 | 92% | <0.001 |
| **Total** | 803 | 2893.249 |  | 3.815 | 100% | <0.001 |
|  |  |  |  |  |  |  |
| **df** degree of freedom , **SS** Sum of Squares, **MS** Mean of Squares, | | | | |  |  |

**Supplementary table S7:** Diversity statistic from protein profiles tested in accession of common vetch (n=381 accessions/ 31 loci)

| **Locus** | **Band Freq.** | **p** | **q** | **Ne** | **H** | **He** | **uHe** |
| --- | --- | --- | --- | --- | --- | --- | --- |
| **A0** | 0.005 | 0.003 | 0.997 | 1.005 | 0.018 | 0.005 | 0.005 |
| **A1** | 0.919 | 0.715 | 0.285 | 1.689 | 0.598 | 0.408 | 0.408 |
| **A1b** | 0.042 | 0.021 | 0.979 | 1.043 | 0.103 | 0.042 | 0.042 |
| **A1c** | 0.068 | 0.035 | 0.965 | 1.072 | 0.151 | 0.067 | 0.067 |
| **A1d** | 0.000 | 0.000 | 1.000 | 1.000 | 0.000 | 0.000 | 0.000 |
| **A2** | 0.921 | 0.719 | 0.281 | 1.677 | 0.594 | 0.404 | 0.404 |
| **A2b** | 0.100 | 0.051 | 0.949 | 1.108 | 0.202 | 0.097 | 0.097 |
| **A4** | 0.039 | 0.020 | 0.980 | 1.041 | 0.098 | 0.039 | 0.039 |
| **A5** | 0.992 | 0.911 | 0.089 | 1.193 | 0.300 | 0.162 | 0.162 |
| **A5b** | 0.037 | 0.019 | 0.981 | 1.038 | 0.092 | 0.036 | 0.036 |
| **A6** | 0.016 | 0.008 | 0.992 | 1.016 | 0.046 | 0.016 | 0.016 |
| **A7** | 0.984 | 0.875 | 0.125 | 1.281 | 0.378 | 0.219 | 0.220 |
| **A11** | 0.441 | 0.252 | 0.748 | 1.606 | 0.565 | 0.377 | 0.378 |
| **A12** | 0.995 | 0.928 | 0.072 | 1.155 | 0.260 | 0.134 | 0.135 |
| **B1** | 0.992 | 0.911 | 0.089 | 1.193 | 0.300 | 0.162 | 0.162 |
| **B2** | 0.323 | 0.177 | 0.823 | 1.411 | 0.467 | 0.291 | 0.292 |
| **B3** | 0.008 | 0.004 | 0.996 | 1.008 | 0.026 | 0.008 | 0.008 |
| **B4** | 0.693 | 0.446 | 0.554 | 1.977 | 0.687 | 0.494 | 0.495 |
| **B5** | 0.911 | 0.701 | 0.299 | 1.721 | 0.610 | 0.419 | 0.420 |
| **B6** | 0.927 | 0.729 | 0.271 | 1.653 | 0.584 | 0.395 | 0.396 |
| **B7** | 0.971 | 0.830 | 0.170 | 1.393 | 0.456 | 0.282 | 0.282 |
| **B8** | 0.751 | 0.501 | 0.499 | 2.000 | 0.693 | 0.500 | 0.501 |
| **B9** | 0.992 | 0.911 | 0.089 | 1.193 | 0.300 | 0.162 | 0.162 |
| **B11** | 0.079 | 0.040 | 0.960 | 1.084 | 0.169 | 0.077 | 0.077 |
| **B12** | 0.024 | 0.012 | 0.988 | 1.024 | 0.064 | 0.023 | 0.024 |
| **B13** | 0.982 | 0.864 | 0.136 | 1.306 | 0.397 | 0.234 | 0.235 |
| **C1** | 0.984 | 0.875 | 0.125 | 1.281 | 0.378 | 0.219 | 0.220 |
| **C2** | 0.032 | 0.016 | 0.984 | 1.032 | 0.082 | 0.031 | 0.031 |
| **C3** | 0.011 | 0.005 | 0.995 | 1.011 | 0.033 | 0.010 | 0.010 |
| **C4** | 0.997 | 0.949 | 0.051 | 1.108 | 0.202 | 0.097 | 0.097 |
| **D1** | 0.974 | 0.838 | 0.162 | 1.373 | 0.443 | 0.272 | 0.272 |
|  |  |  |  |  |  |  |  |
|  |  |  |  |  |  |  |  |
|  | **Band Freq.** | **p** | **q** | **Ne** | **I** | **He** | **uHe** |
| **Mean** | 0.52 | 0.43 | 0.57 | 1.28 | 0.30 | 0.18 | 0.18 |
| **SE** | 0.45 | 0.40 | 0.40 | 0.30 | 0.22 | 0.16 | 0.16 |

**Supplementary table S8:** Descriptors for *Vicia sativa* L., and phenotypic characterization data of the VCC accessions. See Data sheet table 8
